# Protocol for live-cell imaging of mitochondrial dynamics in adult *Drosophila* oenocytes

**DOI:** 10.1016/j.xpro.2026.104370

**Published:** 2026-02-16

**Authors:** Ankur Kumar, Hua Bai

**Affiliations:** 1 Interdisciplinary Genetics and Genomics Program, Iowa State University, Ames, IA 50011, USA; 2 Department of Genetics, Development, and Cell Biology, Iowa State University, Ames, IA 50011, USA

**Keywords:** Cell Biology, Genetics, Microscopy, Model Organisms

## Abstract

Mitochondrial dynamics are essential for cellular homeostasis and can be visualized in adult *Drosophila* oenocytes using live-cell confocal imaging. Here, we present a protocol for live-cell imaging of mitochondrial dynamics in adult *Drosophila* oenocytes. We describe steps for fly preparation, dissection of abdominal cuticle to expose oenocytes, and mounting. We then detail procedures for time-lapse acquisition of mitochondria labeled with mitoGFP. Optimized imaging parameters enable reproducible visualization of mitochondrial morphology stably for longer durations.

## Highlights

• Steps for mounting adult *Drosophila* for live mitochondrial imaging
• Procedures for accessing oenocytes through the cuticle for confocal imaging
• Instructions for visualizing paraquat-induced mitochondrial fission using mitoGFP
• Guidance on quantifying mitochondrial dynamics using Fiji

## Abstract (editor highlights)

Publisher’s note: Undertaking any experimental protocol requires adherence to local institutional guidelines for laboratory safety and ethics.

## Graphical abstract

**Figure:**
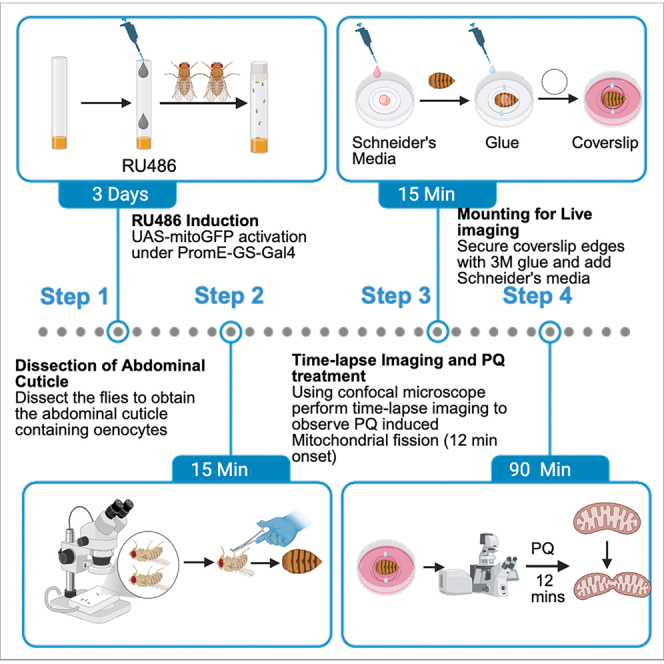

## Before you begin

Mitochondria are highly dynamic organelles whose morphology reflects a balance between fission and fusion. These processes are essential for maintaining cellular homeostasis, and their dysregulation contributes to aging and disease.^1^^,^^2^ While live-cell imaging of mitochondrial dynamics has been successfully demonstrated in Drosophila embryos^3^^,^^4^; and larvae,^5^^,^^6^ there is currently no established protocol for performing live-cell or time-lapse imaging in adult Drosophila oenocytes. Adult tissues present unique challenges, including cuticle opacity, tissue movement, and difficulties in maintaining tissue viability during imaging.

This protocol describes a detailed workflow to prepare adult flies expressing mitoGFP in oenocytes, dissect the abdominal cuticle to expose intact oenocytes, and mount the tissue for stable time-lapse confocal imaging. The method enables visualization of mitochondrial morphology and dynamics for up to 90 minutes under near-physiological conditions. As a demonstration, we show that paraquat (PQ) treatment induces mitochondrial fission in oenocytes, indicating oxidative stress–mediated mitochondrial remodeling. In our associated research, we further used this protocol to compare mitochondrial dynamics between young and old flies, revealing age-associated inhibition of mitochondrial fission.^7^

This protocol focuses on adult oenocytes, hepatocyte-like cells involved in lipid metabolism and detoxification, but the workflow can be adapted to other tissues located beneath or attached to the abdominal cuticle, such as the fat body or abdominal muscles, with minor modifications. Media preparation, RU486 induction, and PQ stock solutions should be completed before beginning dissections and imaging.

### RU486 and GeneSwitch considerations

This protocol employs the PromE-GeneSwitch-GAL4 driver to achieve inducible, adult-specific expression of mitoGFP in oenocytes. Previous studies have shown that RU486 can alter gene expression and mitochondrial function in a tissue- and driver-dependent manner, particularly in thoracic skeletal muscle.^8^ Importantly, PromE-GeneSwitch-GAL4 restricts transgene expression to oenocytes and shows no reported activity in skeletal muscle. Under the induction conditions used here (200 μM RU486 for 3 days), no baseline alterations in oenocyte mitochondrial morphology were observed prior to stressor treatment. Nevertheless, users should be aware that RU486 effects may vary depending on tissue context, driver expression pattern, and induction duration, and appropriate controls are recommended when adapting this protocol to other GeneSwitch drivers or tissues.

### Fly preparation

This protocol uses a custom Drosophila line generated by crossing PromE-GeneSwitch-GAL4 with UAS-mitoGFP enabling RU486-inducible expression of mitoGFP.

PromE-GS-GAL4 is a tissue-specific GeneSwitch driver that restricts mitoGFP expression to adult oenocytes

**Timing: 3–7 days**

1. Prepare fly cultures.
  a. Maintain *Drosophila melanogaster* at 25°C and 60% humidity on standard cornmeal–yeast–agar food.
  b. Collect newly eclosed mixed-sex flies within 24 h, and then separate females and males. Use age-matched, recently mated females for imaging to control for age and mating status.
  c. Monitor fly health daily, ensuring no overcrowding or contamination.
2. Induce mitoGFP expression using the GeneSwitch system. (Applies to PromE-GeneSwitch-GAL4 used in this protocol).
  a. Prepare RU486-supplemented food (200 μM RU486 in standard food).
    ***Note:*** RU486 is required only for GeneSwitch-inducible GAL4 systems such as PromE-GeneSwitch-GAL4. This step is not required for constitutive GAL4 drivers.
    ***Note:*** Prepare RU486 food fresh weekly; store at 4°C protected from light for up to 7 days. Avoid using RU486 food older than 2 weeks due to reduced induction efficiency.
  b. Transfer flies to RU486 food for 3 days to induce mitoGFP expression in oenocytes via the PromE-GeneSwitch-GAL4 driver.
    **CRITICAL:** RU486 can exhibit reproductive toxicity in humans. Handle RU486 using appropriate personal protective equipment (lab coat, nitrile gloves, and mask) and prepare RU486 food in a chemical fume hood. Dispose of waste according to institutional safety guidelines.
    **CRITICAL:** Three days of RU486 feeding provides strong mitoGFP fluorescence while avoiding signal loss from prolonged induction.

### Microscope preparation

**Timing: 30–45 min**

3. Prepare the confocal microscope.
  a. Turn on the confocal microscope (e.g., Olympus FV3000) and allow lasers/electronics to warm up for at least 30 min.
  b. Clean objective lenses with lens paper and cleaning solution.
  c. Calibrate for GFP detection (488 nm excitation, 500–550 nm emission).

**CRITICAL:** Enable autofocus to maintain focus during long time-lapse imaging (up to 90 min).

### Paraquat solution preparation

**Timing: 15–20 min**

4. Prepare PQ stock solutions.
  a. Dissolve paraquat dichloride in Schneider’s Drosophila Medium (DSM) to make 1 M stock solution.
  b. Dilute to working concentrations (600 μM) immediately before use.
  c. Protect all PQ solutions from light and always prepare it fresh on the day of use.

***Note:*** Paraquat (PQ) 1 M stock solution can be stored at 4°C protected from light for 2–4 weeks. Discard stocks older than 4 weeks due to potential degradation and reduced reproducibility.

**CRITICAL:** Paraquat is extremely toxic and can cause pulmonary fibrosis, oxidative injury, and systemic lethality upon exposure. Handle paraquat only inside a certified chemical fume hood using Personal Protective Equipment (PPE), including lab coat, nitrile gloves, and safety mask/eye protection. Dispose of all paraquat waste according to institutional hazardous chemical regulations.

***Alternatives:*** Depending on experimental goals, alternative mitochondrial stressors such as rotenone or antimycin A may be used to induce oxidative stress. These agents require separate optimization and safety evaluation.

### Innovation

This protocol establishes a reproducible workflow for live-cell imaging of mitochondrial dynamics in adult Drosophila oenocytes, which has not previously been available due to cuticle opacity and stability challenges. The innovation lies in optimizing tissue preparation and mounting to stabilize adult abdominal cuticle for up to 60-90 min of imaging while preserving mitochondrial morphology. The protocol also integrates paraquat stimulation to trigger mitochondrial fission in real time, serving as a stress-induced benchmark. Importantly, this approach enabled direct comparison of mitochondrial dynamics between young and old flies in our associated research, revealing age-related inhibition of fission. By expanding live-cell imaging capabilities beyond embryos and larvae, this protocol opens the door for studying aging and stress responses in adult tissues such as oenocytes, fat body, and muscle.

### Institutional permissions

All experiments with *Drosophila melanogaster* were conducted under Iowa State University’s institutional guidelines for invertebrate research. Researchers using this protocol must obtain appropriate approvals in line with their institutional and national regulations.

## Key resources table

**Table:** 

REAGENT or RESOURCE	SOURCE	IDENTIFIER
**Biological samples**		

Adult *Drosophila melanogaster* oenocytes	This study	N/A

**Chemicals, peptides, and recombinant proteins**		

Paraquat (methyl viologen dichloride hydrate)	Fisher scientific	Cat#AC227320050
Mifepristone (RU486)	Cayman Chemical	Cat#10006317
Schneider’s Drosophila Medium (DSM)	Thermo Fisher Scientific	Cat#21720024
FlyNap	Carolina Biological	Cat#173110
3M Instant Adhesives, 0.7 oz Bottle Surface Insensitive Instant Adhesive	Grainger	Cat#29UJ23

**Deposited data**		

	This paper	

**Experimental models: Organisms/strains**		

PromE-GS-Gal4	Gift from Heinrich Jasper	Chatterjee et al.^9^
UAS-mitoGFP	Bloomington Drosophila Stock Center (BDSC)	RRID:BDSC_8442

**Software and algorithms**		

ImageJ/Fiji (v1.54p;NIH)	Schindelin et al.^10^	https://imagej.net/software/fiji/
Mitochondria Analyzer Plugin for ImageJ/fiji (v2.3.1)	Chaudhry et al.^11^	https://github.com/AhsenChaudhry/Mitochondria-Analyzer
GraphPad Prism	GraphPad Software	https://www.graphpad.com/

**Other**		

Olympus FV3000 Confocal Microscope	Olympus Corporation	https://www.olympus-lifescience.com/en/microscopes/confocal/fv3000/
Glass-bottom dishes, 35 mm	MatTek Corporation	Cat#P35G-1.5-14-C
Round glass coverslips, 8 mm diameter, #1.5 thickness	Electron Microscopy Sciences (via Fisher Scientific)	Cat# 50-949-314
Dissection Microscope	Olympus Corporation	SZ61
Scissors	Roboz Surgical Instrument Co., Inc.	B740
Forceps	DUMONT	3C-Antimagnetic-E

## Step-by-step method details

### Dissection of adult *Drosophila* oenocytes

**Timing: 10–15 min**

In this step, you dissect the abdominal cuticle to expose intact oenocytes for imaging. This preparation minimizes tissue damage and ensures oenocyte viability. The procedure is adapted from the oenocyte dissection protocol of,^12^ with modifications to maintain tissue integrity during live confocal imaging.

1. Anesthetize female adult flies (3–5 days old) using FlyNap for 1–2 min.
  a. Confirm immobility under a dissecting microscope.
  b. Transfer flies to a Vaseline-coated dissection dish to stabilize.

***Note:*** Although female adult flies were used in this study, the same dissection and imaging procedure can be applied to male flies.

2. Remove the head and thorax using micro scissors. Quickly add the warm media (25°C), make a small incision at the posterior tip to access the abdominal cavity.

**CRITICAL:** Keep the cuticle submerged in Schneider’s Drosophila Medium throughout dissection to prevent desiccation.

**Figure 1:**
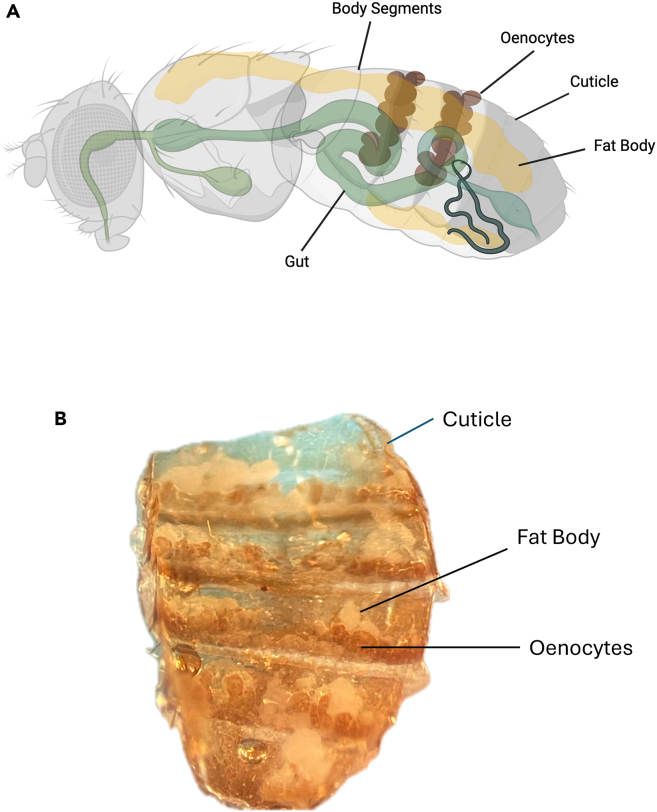
Identification of oenocytes and surrounding abdominal tissues in adult Drosophila for live imaging (A) Schematic illustration of the adult *Drosophila melanogaster* abdomen showing the relative spatial organization of major internal tissues with respect to the abdominal cuticle. Oenocytes are arranged as segmentally repeated clusters positioned directly beneath the inner surface of the cuticle. The fat body lies immediately internal to the oenocytes, while the gut occupies the central abdominal cavity. This organization illustrates why removal of gut and fat body is required during dissection to expose intact oenocytes while preserving their attachment to the cuticle for stable live-cell mitochondrial imaging. (B) Representative dissecting microscope image of an isolated abdominal cuticle preparation showing visible oenocyte clusters and underlying fat body prior to removal. Arrows indicate oenocyte clusters attached to the inner surface of the cuticle.

3. Open the abdominal cuticle along the ventral midline and gently spread it flat.
4. Remove non-oenocyte tissues (gut, fat body, reproductive organs) using fine forceps or low suction (Figure 1A) (Figure 1B).
  a. Keep suction gentle to avoid detaching oenocytes, which remain attached to the inner cuticle.
  b. Trim curved edges of the cuticle to improve flat mounting.
5. Verify oenocyte clusters under a dissecting microscope at ∼20× magnification (Figure 1B).

***Note:*** Oenocytes appear as large polygonal cells in clusters along the cuticle.

### Mounting samples for live imaging

**Timing: 10–15 min**

**Figure 2:**
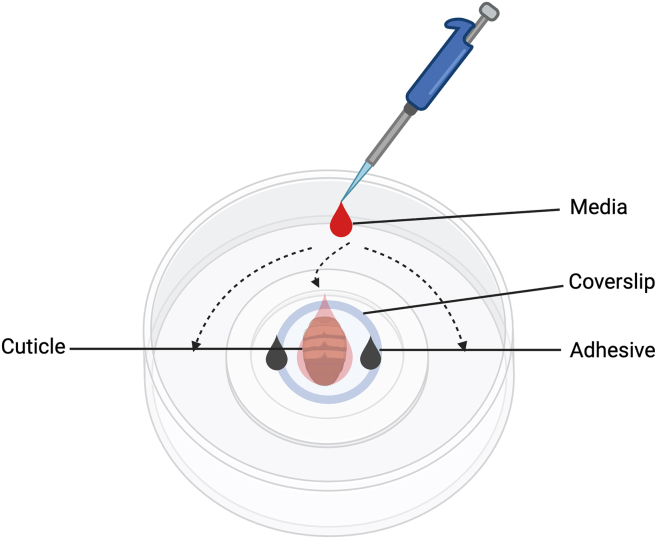
Mounting setup and media flow for live-cell imaging of adult Drosophila oenocytes Schematic representation of the mounting configuration on a glass-bottom dish. The dissected abdominal cuticle is positioned beneath an 8-mm coverslip and secured by two droplets of surface-insensitive cyanoacrylate adhesive placed ∼2–3 mm away from the tissue. Imaging medium is added slowly at the outer edge of the dish, where capillary forces (dashed arrows) guide the medium under the coverslip to fully immerse the tissue without disrupting its position. This arrangement stabilizes the preparation for extended live-cell imaging (up to 90 min) while maintaining tissue viability and minimizing drift.

This step prepares the dissected abdominal cuticle for stable time-lapse imaging of oenocytes under a coverslip in a glass-bottom dish (Figure 2).

6. Prepare the imaging dish.
  a. Clean the glass-bottom dish with 70% ethanol and allow it to air-dry. Ensure no residual ethanol remains before adding medium (Figure 3A).

**Figure 3:**
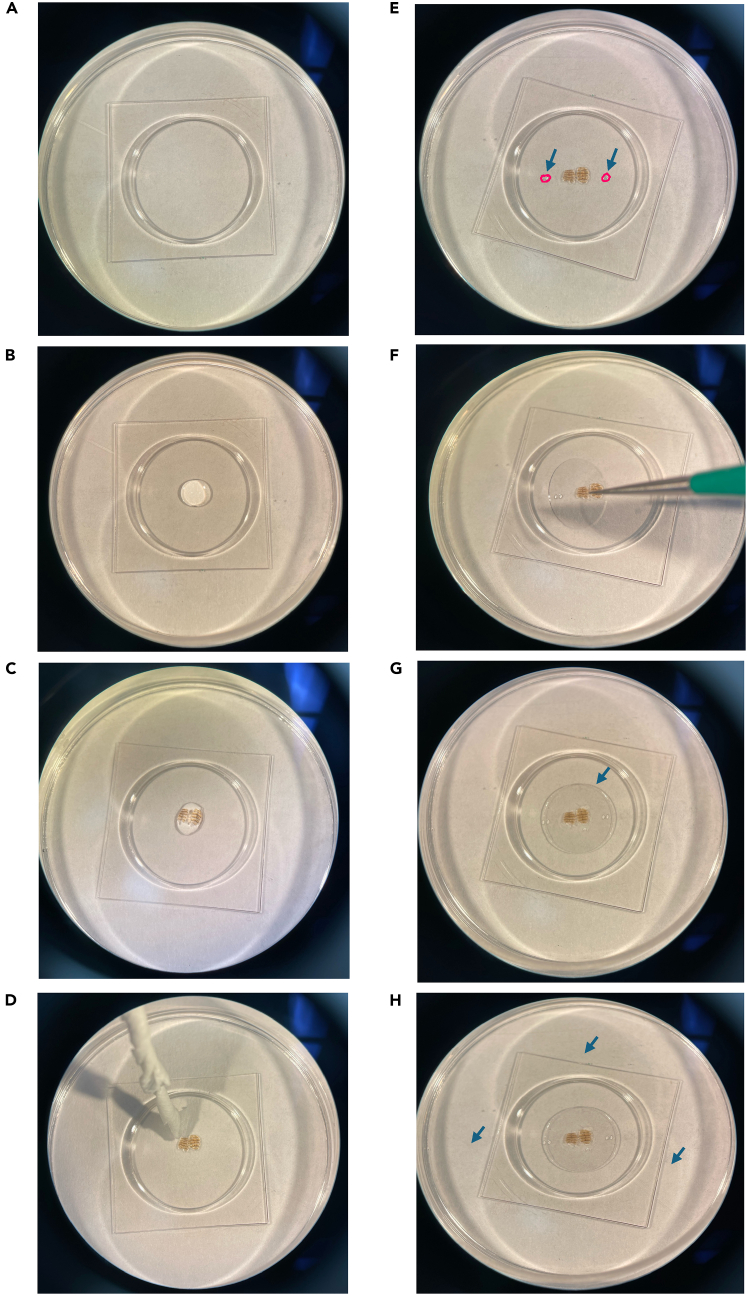
Visualization of mounting setup for live-cell imaging of adult Drosophila cuticle Stepwise visualization of the mounting process for live imaging of oenocytes on the abdominal cuticle. (A) Clean glass-bottom dish prepared for mounting. (B) Addition of ∼10 μL Schneider’s Drosophila Medium (DSM) to the center of the dish. (C) Placement of the dissected abdominal cuticle in DSM with oenocytes facing downward. (D) Gentle removal of excess medium using a Kimwipe to flatten the tissue. (E) Application of ∼1 μL 3M instant adhesive (blue arrows) on opposite sides of the cuticle. (F) Positioning of an 8 mm circular coverslip above the cuticle using forceps. (G) Gradual Lowering of the coverslip at a slanted angle to avoid air bubbles formation and uneven pressure. (H) Final mounted preparation after addition media to the cuticle and ∼1 mL DSM along the outer edge of the dish (blue arrows), allowing capillary flow to fully submerge the sample and maintain tissue hydration during time-lapse imaging.
  b. Add ∼10 μL Schneider’s Drosophila Medium (DSM) pre-equilibrated to 25°C at the center of the glass-bottom well (Figure 3B).
7. Position the dissected tissue.
  a. Transfer the dissected abdominal cuticle into the DSM drop using fine forceps, with the oenocytes facing downward (Figure 3C).
  b. Gently remove excess DSM around the tissue using the edge of a Kimwipe to promote flat positioning (Figure 3D).
8. Prepare for coverslip placement.
  a. Estimate the area where the coverslip will be seated and place two small (∼1 μL) drops of 3M surface-insensitive cyanoacrylate glue (e.g., 29UJ23) on opposite sides of the drop, ∼2–3 mm outside the tissue boundary, as indicated by circles and arrows (Figure 3E).
  b. Ensure the glass surface is completely dry before adding glue.
  c. Wait 10–15 s for the glue to become tacky.

**CRITICAL:** If glue is added to a moist surface, it will not adhere properly and can cause the coverslip to drift during imaging.

9. Place and secure the coverslip.
  a. Align an 8 mm circular #1.5 coverslip above the cuticle and lower it gently at a slanted angle to minimize bubble formation (Figure 3F).
  b. Allow the coverslip edges to contact the glue to secure the sample in place. Apply minimal pressure to flatten the cuticle without compressing oenocytes.

**CRITICAL:** Do not let glue come into contact with the Schneider’s Drosophila Medium (DSM) or tissue. Excessive pressure can damage oenocytes or distort mitochondrial morphology.

**CRITICAL:** Lower the coverslip gradually from one side to allow trapped air to escape and to prevent bubble formation under the tissue.

***Note:*** The seal should prevent sample drift during imaging while permitting sufficient medium diffusion to maintain oenocyte viability for up to 90 min.

10. Add imaging medium.
  a. Using a 10 μL pipette, gently add a small volume of Schneider’s Drosophila Medium (DSM) beneath the coverslip edge to ensure the tissue is fully wetted without disturbing its position, indicated by an arrow (Figure 3G).
  b. Slowly add 1–2 mL of Schneider’s Drosophila Medium (DSM) along the outer edges of the glass-bottom dish to fully submerge the sample, indicated by arrows (Figure 3H).

**CRITICAL:** Add medium gradually along the rim to avoid disturbing the coverslip seal or introducing air bubbles beneath it.

***Note:*** Proper submersion and balanced sealing maintain oenocyte viability and stable imaging conditions for up to 90 min.

### Live confocal imaging of mitochondrial dynamics

**Timing: 60–90 min**

In this step, the mounted oenocyte preparation is imaged using confocal microscopy to capture mitochondrial morphology and dynamics over time. mitoGFP is used for visualization, and paraquat (PQ) can be added during imaging to induce mitochondrial fission in real-time. Paraquat is well known for inducing oxidative stress.^13^ Mitochondrial fission is induced by PQ treatment and concentration used here is determined based on previous studies and trials.^14^ PQ was added after 12 minutes to ensure that tissue is stabilized.

11. Place the mounted dish on the stage of the confocal microscope (e.g., Olympus FV3000).
  a. Remove the dish lid to allow drug addition during imaging.
  b. Secure the dish holder to minimize vibration.
12. Locate oenocytes at 10× magnification to confirm mitoGFP expression and select regions with strong fluorescence.
13. Switch to a 40× objective for imaging.
  a. Set zoom to 3–5× to visualize individual mitochondria.
  b. Adjust laser power to 0.5%–3% to minimize (488 nm excitation; 500–550 nm emission) photobleaching.
14. Enables autofocus to maintain a stable focal plane during the time-lapse.

**CRITICAL:** A flat cuticle and balanced coverslip seal are required for consistent autofocus performance.

***Note:*** Select the single focal plane that shows the highest mitochondrial contrast and sharpest mitoGFP signal across multiple oenocytes before initiating time-lapse acquisition. This plane typically corresponds to the equatorial section of the oenocyte layer where mitochondrial networks are most clearly resolved.

15. Configure the time-lapse acquisition.
  a. Capture images every 2 min for up to 60+ min.
  b. Keep pinhole at 1 Airy unit for optimal signal-to-noise.
  c. Use line averaging (2–3×) if background noise is high.

***Note:*** Although Z-stack imaging can be used to obtain volumetric mitochondrial morphology, it significantly increases photobleaching, acquisition time, and axial drift during prolonged live imaging. Therefore, single-plane imaging was selected in this protocol to maximize temporal resolution and maintain mitochondrial signal stability over long time courses.

16. Begin baseline imaging for 12 min (six cycles) to establish control mitochondrial morphology.
17. Introduce PQ or control medium by capillary exchange.
  a. After 12 min of baseline imaging, slowly add 1–2 mL of 600 μM paraquat (PQ) solution to the outer edge of the glass-bottom dish using a pipette to achieve a final concentration of 300 μM PQ.
  b. Add the solution along the dish wall, not directly at the coverslip edge, to allow capillary-driven media exchange beneath the coverslip without disturbing the tissue.

**CRITICAL:** Dispense PQ slowly to prevent hydrodynamic disturbance, sample drift, or autofocus disruption.

18. Control condition (no PQ).

For untreated control samples, add the same volume (1–2 mL) of fresh DSM at the dish edge using the identical capillary exchange method described above.

**CRITICAL:** Volume-matched DSM addition ensures that any observed morphological changes are due to PQ exposure and not mechanical perturbation.

19. Continue imaging for up to 60 or more mins, recording mitochondrial dynamics before and after PQ addition.

***Note:*** Mitochondria typically transition from tubular to fragmented morphology within 30–60 min after PQ treatment.

20. Save image stacks as.life or.oib/.oir files (depending on microscope software) for downstream quantification.
21. After imaging, discard samples following institutional chemical safety guidelines for PQ.

### Image processing and quantification of mitochondrial number

**Timing: 30–45 min per dataset**

Analysis of mitochondrial number during live-cell imaging

This step describes the in-silico analysis of mitochondrial numbers over time using the Mitochondria Analyzer plugin (v2.3.1)^11^ implemented in Fiji/ImageJ (v1.54p;NIH). Confocal time-lapse stacks are preprocessed and analyzed to quantify mitochondrial counts per frame, which provide a readout of fission dynamics. Representative images and videos were processed with Gaussian filter.

22. Open the confocal time-lapse stack in Fiji/ImageJ (Figure 4A).

**Figure 4:**
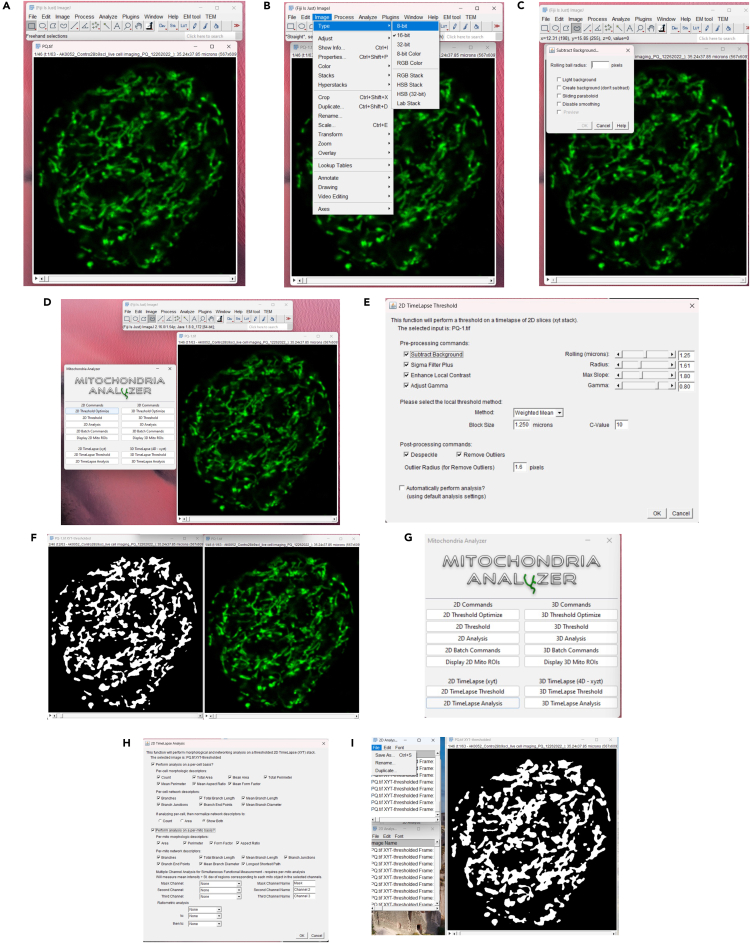
Image Processing and Quantification Workflow Using Fiji and Mitochondria Analyzer (A) Representative raw confocal mitoGFP image from adult Drosophila oenocytes used for mitochondrial quantification. (B) Conversion of the time-lapse image stack to 8-bit format (Image → Type → 8-bit), required for downstream thresholding and plugin compatibility. (C) Background subtraction using the Rolling Ball algorithm (Process → Subtract Background), applied uniformly across all datasets to improve mitochondrial contrast. (D) Launching the Mitochondria Analyzer (v2.3.1) plugin in Fiji/ImageJ (v1.54p), showing the main interface and available analysis modules. (E) Configuration window for the 2D TimeLapse Thresholding module, displaying preprocessing, local thresholding, and post-processing options used to segment individual mitochondria. (F) Output of the segmentation step: left, thresholded binary image showing individual mitochondria detected by the plugin; right, corresponding original frame for visual comparison. Segmentation accuracy was validated by comparing the binary output with the raw image before quantification. (G) Main interface of the Mitochondria Analyzer plugin (v2.3.1) in Fiji/ImageJ, showing available commands for 2D and 3D analyses, including modules specific for time-lapse datasets (2D TimeLapse Threshold; 2D TimeLapse Analysis). (H) Parameter configuration window for 2D TimeLapse Analysis used to extract mitochondrial metrics from thresholded stacks. Only the “Number of mitochondria per frame” output was used in this protocol; other descriptors (e.g., branch count, form factor) were not included. (I) Example of a processed dataset showing the thresholded binary output (left) and the corresponding raw mitoGFP frame (right). Each white object represents a segmented mitochondrion. The plugin outputs mitochondrial counts for each time point, which are exported as spreadsheets for downstream plotting and comparison between untreated and PQ-treated samples.
23. Convert the image stack to 8-bit format (Image → Type → 8-bit) (Figure 4B).
24. Perform background subtraction (Process → Subtract Background) using a rolling ball radius optimized for mitochondrial signal (Figure 4C).

**CRITICAL:** Use the same subtraction parameters for all samples to ensure comparability.

25. Launch the Mitochondria Analyzer plugin in Fiji/ImageJ (Figure 4D).

***Note:*** This protocol analyzes the entire field of view, and therefore all confocal images must be acquired with a consistent frame size, magnification, and zoom.

If a region of interest (ROI) is used, it must be applied identically across all frames and should only be chosen to exclude non-oenocyte regions.

26. Apply 2D TimeLapse Thresholding to segment mitochondria from background (Figure 4E).
27. Compare the thresholded image to the original to confirm accurate segmentation (Figure 4F).
28. Re-adjust the threshold if mitochondria appear over-segmented (merged) or under-segmented (lost).
29. Run 2D TimeLapse Analysis to generate mitochondrial number outputs for each frame (Figures 4G and 4H).
30. Export the raw data as an Excel file and save it in the designated analysis folder (Figure 4I).
31. From the output, extract only the “Number of mitochondria” per frame values.
32. Plot mitochondrial numbers over time to assess fission dynamics under untreated and paraquat (PQ)-treated conditions.

***Note:*** Baseline mitochondrial counts vary between imaging fields due to differences in oenocyte cluster size and cell number; therefore, analyses focus on relative changes over time within each sample rather than absolute starting values.

***Note:*** While the Mitochondria Analyzer plugin generates multiple morphological descriptors, only mitochondrial numbers were analyzed in this protocol.

## Expected outcomes

**Figure 5:**
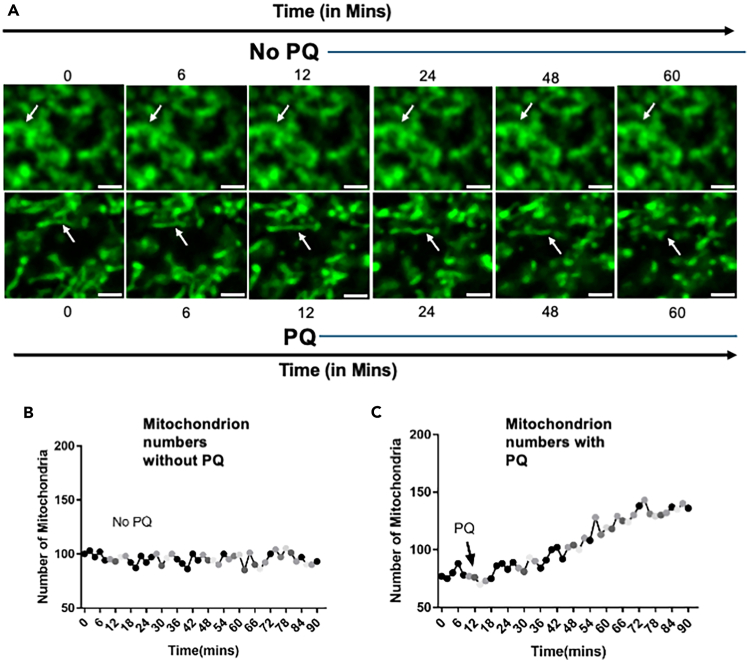
Live-cell imaging of PQ-induced mitochondrial fission in adult Drosophila oenocytes (A) Time-lapse confocal images of mitochondria labeled with mitoGFP in oenocytes. Top: untreated controls (No PQ) show stable, tubular mitochondria with little change over 60 min (Methods Video S1). Bottom: PQ treatment induces progressive mitochondrial fragmentation over time (Methods Video S2), visible as shorter and more numerous structures. White arrows indicate representative mitochondria undergoing dynamic changes. Scale bars, 2 μm. (B) Quantification of mitochondrial numbers without PQ treatment shows minimal fluctuation over 90 min. (C) Quantification of mitochondrial numbers with PQ treatment demonstrates a time-dependent increase in mitochondrial fragmentation, with a marked rise after 30–60 min and continuing through 90 min.

This protocol enables visualization of mitochondrial dynamics in adult Drosophila oenocytes using mitoGFP and confocal microscopy. Under basal conditions, mitochondria appear tubular and interconnected (Figure 5; Methods Video S1). Upon paraquat (PQ) addition, fragmentation occurs within 30–60 min, resulting in shorter and more numerous mitochondria (Figure 5; Methods Video S2). The optimized mounting ensures stable imaging with minimal photobleaching. These outcomes can be reproduced consistently across individual flies. In our associated work (Kumar et al., preprint), we further applied this protocol to compare young and old flies, demonstrating age-associated inhibition of PQ-induced mitochondrial fission.

Methods Video S1. Basal mitochondrial dynamics in adult Drosophila oenocytes (related to Figure 4 and step 17) Time-lapse confocal imaging of PromE > mitoGFP oenocytes under normal conditions. Images were acquired every 2 min for 90 min at 40× magnification. Mitochondria exhibit predominantly elongated morphology with occasional transient fission and fusion events. Tissue remained viable throughout imaging. Scale bar, 5 μm.

Methods Video S2. Paraquat-induced mitochondrial fission in adult Drosophila oenocytes (related to Figure 4 and step 18) Time-lapse confocal imaging of PromE > mitoGFP oenocytes after paraquat (PQ) feeding. Images were acquired every 2 min for 90 min at 40× magnification. PQ exposure induces progressive mitochondrial fragmentation and increased fission frequency over time. Scale bar, 5 μm.

### Image presentation

Confocal images were processed in Fiji/ImageJ after quantification for improved visualization. A Gaussian blur filter (σ = 2.0 pixel) was applied uniformly to all frames to reduce background noise and enhance contrast. This filtering step was not used for quantitative analysis; all quantification was performed on unfiltered raw image stacks.

## Limitations

This protocol provides a reproducible workflow for live-cell imaging of mitochondrial dynamics in adult Drosophila oenocytes; however, several limitations should be considered.

First, tissue viability under the coverslip is limited to approximately 90 min, which restricts the duration of imaging. Extended imaging increases the likelihood of photobleaching, tissue drift, and mitochondrial signal loss.

Second, the protocol is optimized for oenocytes, which are located just beneath the abdominal cuticle and can be stably imaged after dissection. Deeper tissues such as heart, muscle, or neurons pose additional challenges due to cuticular opacity and limited optical penetration. Imaging these tissues would require additional modifications, such as tissue clearing or multiphoton imaging.

Third, mitochondrial dynamics in this protocol are visualized using RU486-inducible GeneSwitch-driven mitoGFP expression. While this system provides precise temporal and tissue-specific control, RU486 has been reported to influence mitochondrial gene expression in certain tissues, particularly skeletal muscle. Although such effects have not been reported in adult oenocytes, users should be aware of this limitation and consider appropriate controls or alternative drivers when applying the protocol to other tissues.

Fourth, the paraquat (PQ)–induced fission assay models oxidative stress–driven mitochondrial dynamics but may not fully represent physiological fission regulation under normal metabolic conditions.

Finally, quantitative analysis in this protocol focuses on mitochondrial number per frame as a readout of fission dynamics. Oenocyte clusters vary in size and cell number leading to differences in baseline mitochondrial counts between samples. Additional morphometric parameters (e.g., branch length, network connectivity, aspect ratio) can be extracted using the Mitochondria Analyzer plugin but were not included in the present workflow.

## Troubleshooting

### Problem 1

MitoGFP signal is weak or inconsistent between flies (Step 3 – Fly preparation).

### Potential solution

• Confirm that flies were kept on RU486 food for 3 days, which in our hands gives maximal mitoGFP signal with preserved oenocyte morphology. Prolonged induction (≥5 days) can reduce fluorescence, likely due to GeneSwitch desensitization.
• When establishing a new driver or genotype, we recommend a pilot time-course (e.g., 1, 3, 5 days of RU486 feeding) to determine the shortest induction that yields strong mitoGFP signal without altering oenocyte morphology.
• Verify the genotype and that the GeneSwitch GAL4 driver is present.

### Problem 2

Tissue drifts or moves during time-lapse imaging (Step 8–9 – Mounting samples).

### Potential solution

Apply minimal pressure when placing the coverslip and secure it evenly with small drops of glue at multiple points. Avoid excess glue, which can seep into DSM. If drift persists, make sure you are allowing the coverslip to stick properly before adding the DSM.

### Problem 3

Rapid photobleaching of mitoGFP during time-lapse (Step 13–14 – Confocal imaging setup).

### Potential solution

Photobleaching can occur in long time-lapse acquisitions due to prolonged illumination of fluorescent proteins such as mitoGFP. To minimize bleaching, reduce laser power and detector gain, increase the interval between frames (e.g., from 2 min to 5 min), and use line averaging rather than long exposure times. Allow the laser to warm up prior to imaging for illumination stability.

Additionally, ensure minimal pressure on the coverslip during mounting, as compression increases scattering and accelerates photobleaching.

Photobleaching behavior and strategies for preventing it are well-documented in fluorescence imaging literature,^15^ and Fiji provides photobleaching-correction tools if required.^16^

### Problem 4

PQ does not induce observable fission within 60–90 min (Step 17 – PQ addition).

### Potential solution

Verify PQ stock is fresh and prepared in Schneider’s medium. Use concentrations validated in previous experiments. Ensure baseline imaging (12 min) was captured before PQ addition to compare.

### Problem 5

Loss of focus or Z-drift during long-term imaging (Step 14 – Maintaining focus during imaging).

### Potential solution

Use the confocal microscope’s Z-drift compensation or autofocus if available. If unavailable, select thicker tissues with stable adhesion, and reduce imaging duration to ≤60 min.

### Problem 6

Over- or under-segmentation of mitochondria in ImageJ (Quantification Step 5–7 – ImageJ analysis).

### Potential solution

Optimize thresholding in the Mitochondria Analyzer plugin by testing different settings and comparing to raw images. Perform background subtraction before thresholding. Exclude frames with motion blur or poor signal-to-noise ratio.

### Problem 7

Coverslip detaches during long imaging (Steps 8–9 – Mounting samples).

### Potential solution

If glue adhesion weakens during imaging, the dish may have residual medium on the glass before sealing. Ensure the area where glue is applied is dry before adding the coverslip. Alternatively, use freshly opened 3M surface-insensitive adhesive and pre-warm the dish to room temperature to accelerate curing.

### Problem 8

Air bubbles appear under the coverslip after placement (Step 9).

### Potential solution

Lower the coverslip from one side at a shallow angle to displace air. If bubbles persist, gently tap the dish edge or use a fine needle to release trapped air before sealing. Avoid strong airflow or vibrations during glue curing.

### Problem 9

Tissue dries before mounting (Steps 6–7).

### Potential solution

Limit air exposure of dissected cuticles to ≤ 1 min. Always keep a small DSM droplet on the tissue during transfer.

### Problem 10

PQ diffuses unevenly under the coverslip (Step 17).

### Potential solution

Introduce PQ solution slowly along one edge of the dish using a fine-tip pipette. Capillary flow will distribute it uniformly within 1–2 min. Do not pipette directly over the tissue.

## Resource availability

### Lead contact

Further information and requests for resources and reagents should be directed to and will be fulfilled by the lead contact, Hua Bai (hbai@iastate.edu).

### Technical contact

Technical questions on executing this protocol should be directed to the technical contact, Ankur Kumar (ankurk@iastate.edu).

### Materials availability

The fly stocks are available from the lead contact upon reasonable request. All other *Drosophila* strains and reagents used in this protocol are available from public repositories such as the Bloomington Drosophila Stock Center (BDSC).

### Data and code availability

The protocol includes representative imaging data and quantification workflows. Raw imaging files and ImageJ analysis pipelines are available from the lead contact upon request. No custom code was developed for this study.

## Acknowledgments

This work was supported by the National Science Foundation (NSF) CAREER award 2046984 and the National Institutes of Health (NIH) grant R01AG058741 to H.B. We thank the Bloomington Drosophila Stock Center for fly stocks. We are also grateful to members of the Bai lab for valuable discussions and feedback during protocol development. The graphical abstract and other figures were created using BioRender.com with a lab-based academic subscription.

## Author contributions

A.K. designed and performed the experiments, developed the live imaging workflow, and drafted the manuscript. H.B. supervised the project, provided conceptual guidance, and revised the manuscript. Both authors read and approved the final version of the protocol.

## Declaration of interests

The authors declare no competing interests.
